# Repression of Floral Meristem Fate Is Crucial in Shaping Tomato Inflorescence

**DOI:** 10.1371/journal.pone.0031096

**Published:** 2012-02-07

**Authors:** Johanna Thouet, Muriel Quinet, Stanley Lutts, Jean-Marie Kinet, Claire Périlleux

**Affiliations:** 1 Laboratory of Plant Physiology, Department of Life Sciences, University of Liège, Liège, Belgium; 2 Groupe de Recherche en Physiologie Végétale, Earth and Life Institute, Université Catholique de Louvain, Louvain-la-Neuve, Belgium; Instituto de Biología Molecular y Celular de Plantas, Spain

## Abstract

Tomato is an important crop and hence there is a great interest in understanding the genetic basis of its flowering. Several genes have been identified by mutations and we constructed a set of novel double mutants to understand how these genes interact to shape the inflorescence. It was previously suggested that the branching of the tomato inflorescence depends on the gradual transition from inflorescence meristem (IM) to flower meristem (FM): the extension of this time window allows IM to branch, as seen in the *compound inflorescence* (*s*) and *falsiflora* (*fa*) mutants that are impaired in FM maturation. We report here that *JOINTLESS* (*J*), which encodes a MADS-box protein of the same clade than SHORT VEGETATIVE PHASE (SVP) and AGAMOUS LIKE 24 (AGL24) in *Arabidopsis*, interferes with this timing and delays FM maturation, therefore promoting IM fate. This was inferred from the fact that *j* mutation suppresses the high branching inflorescence phenotype of *s* and *fa* mutants and was further supported by the expression pattern of *J*, which is expressed more strongly in IM than in FM. Most interestingly, *FA* - the orthologue of the *Arabidopsis LEAFY* (*LFY*) gene - shows the complementary expression pattern and is more active in FM than in IM. Loss of *J* function causes premature termination of flower formation in the inflorescence and its reversion to a vegetative program. This phenotype is enhanced in the absence of systemic florigenic protein, encoded by the *SINGLE FLOWER TRUSS* (*SFT*) gene, the tomato orthologue of *FLOWERING LOCUS T* (*FT*). These results suggest that the formation of an inflorescence in tomato requires the interaction of J and a target of SFT in the meristem, for repressing *FA* activity and FM fate in the IM.

## Introduction

Flowering is an important process that determines fruit and seed production in Angiosperms. Most knowledge of its genetic control comes from studies in *Arabidopsis*, a facultative long-day plant which exhibits monopodial growth (reviewed in [1]–[3]). Upon floral transition, the shoot apical meristem (SAM) switches from leaf production to inflorescence meristem (IM) fate and initiates flower meristems (FM) on its flanks. Several environmental and developmental signalling pathways which trigger the floral transition of the SAM have been disclosed; they converge on the transcriptional regulation of two major “integrator genes”, *FLOWERING LOCUS T* (*FT*) and *SUPPRESSOR OF OVEREXPRESSION OF CONSTANS 1* (*SOC1*), which in turn activate the FM identity genes *LEAFY* (*LFY*) and *APETALA1* (*AP1*).

FT was identified as a major output of the photoperiodic pathway that promotes flowering in *Arabidopsis* by the extension of daylength; the FT protein is synthesized in the leaves, travels through the phloem towards the SAM where it then interacts with the bZIP transcription factor FD to activate *AP1* (reviewed in [4]). FT also activates *SOC1*, which together with *AGAMOUS LIKE 24* (*AGL24*) in the SAM, up-regulates *LFY* [5], [6].

The activation of *LFY* and *AP1* is necessary to determine that the lateral primordia on the SAM develop as flowers rather than shoots. Both *lfy* and *ap1* single mutants produce lateral shoots intermediate between vegetative and floral while in *lfy∶ap1* double mutants, lateral primordia develop as vegetative shoots [7]. *LFY* and *AP1* initiate a cascade of changes in gene expression leading to the specification of the floral organ whorls and this requires tight regulation in space and time. Firstly, the activation of *LFY* and *AP1* has to be restricted to the FMs. Maintenance of IM identity in the central dome of the *Arabidopsis* SAM is guaranteed by the expression of *TERMINAL FLOWER 1* (*TFL1*) and *AGL24* that repress the expression of *LFY* and *AP1* while LFY and AP1 inhibit the expression of *TFL1* and *AGL24* in the FM [8], [9]. Secondly, premature differentiation must be avoided in the FM to allow formation of a sufficient number of stem cells before activation of the floral organ identity genes. This involves the combined activity of the MADS-box flowering time genes *SHORT VEGETATIVE PHASE* (*SVP*), *AGL24* and *SOC1* that repress the expression of the transcription factor *SEPALLATA3* (*SEP3*) [10]. This inhibition is relieved by AP1 which, once activated in the FM, directly represses *SVP*, *AGL24* and *SOC1*, so marking floral commitment [11]. Finally, the identity of each whorl of floral organs is specified by the combinatorial action of homeotic genes of class A, B, C and E, expressed in discrete regions of the developing flower (reviewed in [12]). *LFY* activates various floral homeotic genes in combination with specific co-regulators [13]; the targets of LFY include *AP1* which plays a dual role in promoting the initial FM identity and acting as a class A gene to control formation of sepals and petals [14].

Conservation of *Arabidopsis* flowering genes has been shown in many species. This is the case in tomato (*Solanum lycopersicum*), where mutants have been used in both genetic studies and breeding for decades (reviewed in [15]–[17]). Yet further experiments are required for bridging the gap between tomato genes and their exact function because tomato shows several peculiarities when compared with *Arabidopsis*. Firstly, floral transition of modern cultivars is mostly autonomous, as in many crops, and is accelerated by high light availability [18]. Secondly, tomato has a sympodial growth habit; after the floral transition of the SAM, shoot growth is taken over by the axillary meristem of the last leaf, the sympodial meristem (SYM), whose outgrowth is boosted and displaces the first inflorescence laterally. The SYM forms a sympodial segment composed of a few leaves before flowering itself. The process is iterated so that the tomato shoot remains indeterminate, consisting of one initial segment and successive sympodial units. While the initial segment initiates a variable number of leaves, dependant on the time of first floral transition, the sympodial units most often count 3 leaves. The third characteristic of tomato is that it generates a few-flowered inflorescence organized in a zigzag pattern. This structure has been described in contrasting ways, sometimes with confusing terminology as recently reviewed [19], but clearly develops in a different way to the *Arabidopsis* inflorescence. In this paper we adhere to the view that, at floral transition, the SAM of tomato forms a FM and a lateral meristem arises adjacently. This lateral (sympodial) meristem is commonly called IM since it builds the inflorescence by forming the second FM and initiating another lateral IM, and so on.

A few tomato mutants have been characterized at the molecular level, leading to the identification of orthologues to *Arabidopsis* flowering genes (Table 1). This is the case for *SINGLE FLOWER TRUSS* (*SFT*), that is the orthologue of *Arabidopsis FT*, and by the same token, encodes a mobile florigenic protein [20], [21]. The *sft* mutants are late flowering and produce inflorescences that are reduced to one or a few flowers and revert to vegetative functioning [20]–[23].

**Table 1:** Some mutations affecting flowering in tomato.

Mutant	Phenotype	Isolated gene	*Arabidopsis* homologue	References
*single flower truss* (*sft*)	Late flowering. Inflorescence composed by a single flower or reverting to leaf production	*SFT*	*FLOWERING LOCUS T* (*FT*)	[20]–[23]
*jointless* (*j*)	Inflorescence producing a few flowers, then reverting to leaf initiation. Flowers lack pedicel abscision zone.	*J*	*SHORT VEGETATIVE PHASE* (*SVP*) *AGAMOUS-LIKE 24* (*AGL24*)	[23], [24], [26]
*falsiflora* (*fa*)	Late flowering. Highly branched inflorescence containing leafy shoots and cauliflower-like masses of meristematic tissue	*FA*	*LEAFY* (*LFY*)	[27], [28]
*anantha* (*an*)	Highly branched inflorescence showing cauliflower-like masses of meristematic tissue	*AN*	*UNUSUAL FLORAL ORGAN* (*UFO*)	[28], [29]
*compound inflorescence* (*s*)	Highly branched inflorescence with normal flowers	*S*	*WUSHEL HOMEOBOX 9* (*WOX9*)	[23], [29]

*JOINTLESS* is a MADS-box gene which belongs to the same clade as the *Arabidopsis* flowering time genes *SVP* and *AGL24* [24]. The *j* mutant was originally selected because of the absence of pedicel abscission zone [25] and is characterized, like *sft*, by its inflorescence reverting to leaf initiation after formation of a few flowers [23], [26].

*FALSIFLORA* is orthologous to the FM identity gene *LFY* [27]. Consistent with conservation of the FM identity function of *LFY*, the *fa* mutants produce inflorescences made of leafy shoots. These inflorescences are also highly branched and contain clumps of proliferating meristems [27], [28]. The lack of flowers and over-production of meristems are reminiscent of the cauliflower-like phenotype of the *anantha* (*an*) mutant [28]. *AN* is orthologous to *UNUSUAL FLORAL ORGANS* (*UFO*) [29], which functions as a transcriptional co-factor of LFY in *Arabidopsis* [30]. In tomato, the *AN* gene acts downstream of *FA* and is expressed in FM [28], [29]. The complexity of the *an* inflorescence was explained by a gradual transition of IM to FM: meristems that fail to “mature” into FM continue to produce other meristems and branch [29]. In the *compound inflorescence* (*s*) mutant, maturation of FM (and expression of *AN*) is delayed and the inflorescence is highly branched, but eventually bears up to 200 fertile flowers [23], [29]. Therefore, temporal regulation of floral fate appears critical in shaping the tomato inflorescence [31]. The *S* gene encodes a WUSCHEL-homeobox (WOX) transcription factor. During initiation of the inflorescence, *S* is transiently expressed in incipient IM while *AN* is expressed in early FM shortly after downregulation of *S* [29].

It is clear from this survey that a genetic network is involved in the architecture of the inflorescence in tomato. However, the emerging view is still fragmented because most functional analyses concern single mutations affecting either inflorescence or flower fate. Because classical epistasis experiments would contribute to decipher the pathways, we constructed a set of novel double mutants which include all possible combinations of the mutations in the genes *SFT*, *J*, *FA* and *S* (Table 2). A careful analysis of their phenotypes allowed us to suggest a genetic model on the role of *J* and *SFT* in the specification of IM identity.

**Table 2:** Tomato double mutants produced and analysed in this study.

Single mutation	Second mutation	Interaction		Reference
		Flowering time	Inflorescence	
*fa*	*sft*	synergistic	additive	[22], this work
*j*	*fa*	synergistic	additive	This work
*j*	*s*	synergistic	*j* epistatic	This work
*j*	*sft*	s*ft* epistatic	additive	This work
*s*	*fa*	synergistic	*fa* epistatic	This work
*s*	*sft*	*sft* epistatic	additive	[29], this work

## Results

### Flowering time

**Figure 1:**
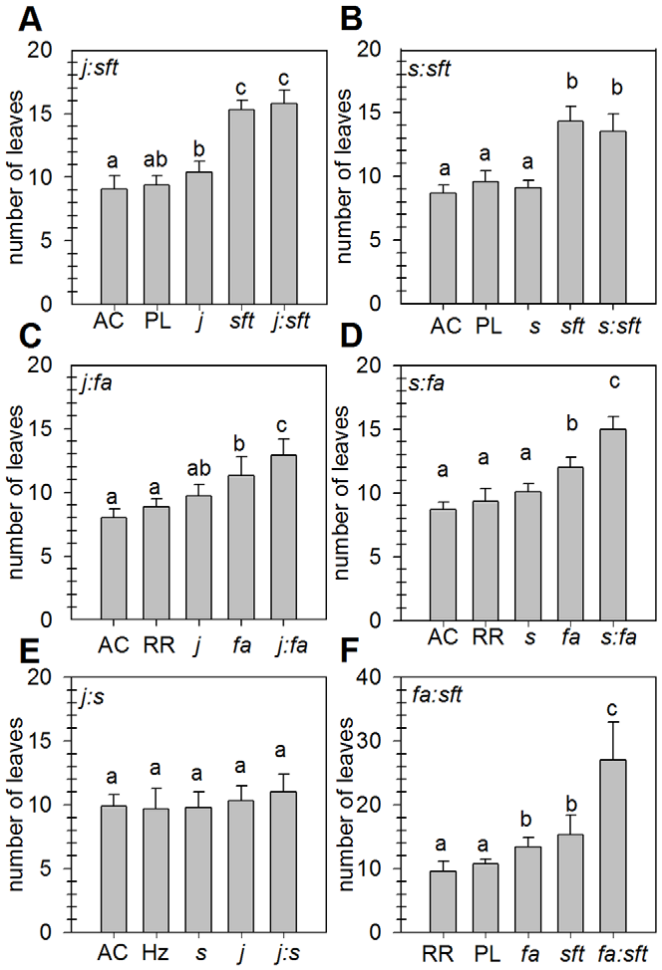
Flowering time (expressed as number of leaves below the first inflorescence) of tomato double mutants. (A) *j∶sft*; (B) *s∶sft*; (C) *j∶fa*; (D) *s∶fa*; (E) *j∶s*; and (F) *fa∶sft*. Values followed by a same letter (a, b, or c) are not statistically different (P<0.05). Genotype abbreviations: AC, Ailsa Craig WT; *fa*, *falsiflora*; Hz, Heinz WT; *j, jointless*; Pl, Platense WT; RR, Rheinlands Rhum WT; *s*, *compound inflorescence*; *sft*, *single flower truss*. The *j* mutant is in AC background in A and C, in Hz background in E.

Among the single mutants used in this study, *sft* and *fa* showed a retardation of flowering of the initial shoot segment while *s* and *j* had no or little effect (Figure 1), as previously shown [21]–[23], [27]. The *sft* and *fa* parents conferred late flowering to the double mutants obtained by crosses with *j* or *s; sft* was epistatic to both *j* and *s* (Figure 1AB, Table 2) while the late flowering phenotype of *fa* was enhanced by *j* and *s*, suggesting synergistic effects (Figure 1CD). Although the *s* and *j* mutations did not markedly affect flowering time alone, the *j∶s* double mutant always produced one more leaf than the parental mutants (Figure 1E).

The double *fa∶sft* mutant showed a very strong delay in flowering, reflecting a synergistic effect of *fa* and *sft* mutations (Figure 1F). Molinero-Rosales *et al.* previously reported that the *fa∶sft* double mutant produced over 100 leaves and did not flower, indicating that *FA* and *SFT* act in two parallel pathways that are both necessary to promote flowering in tomato [22]. Although the phenotype of our *fa∶sft* double mutant was less severe (in our growing conditions, at least) the same conclusion can be inferred from our analysis and is also consistent with the report that in *Arabidopsis*, when combined, mutations in *LFY* and *FT* completely suppress flowering [32].

### Inflorescence architecture

**Figure 2:**
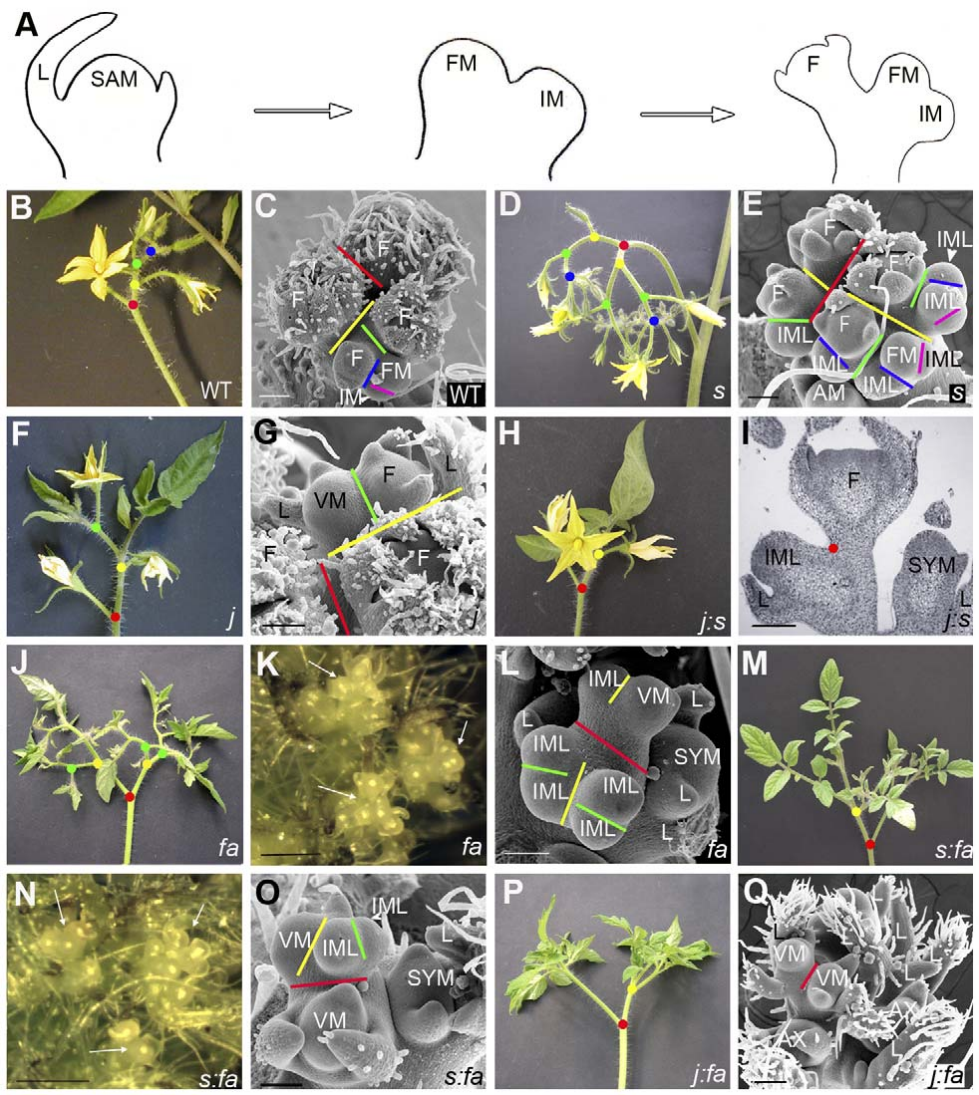
Inflorescence phenotype of tomato mutants. (A–C) Ailsa Craig WT; (D) and (E) *s* mutant; (F) and (G) *j* mutant; (H) and (I) *j∶s* double mutant; (J–L) *fa* mutant; (M–O) *s∶fa* double mutant; (P) and (Q) *j∶fa* double mutant. In microscopic pictures, colour bars show the clefts that occurred sequentially when new meristems were initiated to build-up the inflorescence. Red: 1^st^; yellow: 2^nd^; green: 3^rd^; blue: 4^th^; purple: 5^th^. Dots of same colours show the corresponding branching in macroscopic inflorescence pictures. Pictures K and N zoom in J and M, respectively, to show clumps of IMLs in the inflorescences (arrows). Genotype abbreviations: AC, Ailsa Craig WT; *fa*, *falsiflora*; *j*, *jointless*; *s*, *compound inflorescence*. Annotations: AX, axillary meristem; F, flower; FM, flower meristem; IM, inflorescence meristem; IML, IM-like; L, leaf; SAM, shoot apical meristem; SYM, sympodial meristem; VM, vegetative meristem. Bars = 100 µm except in K and N where bars = 1 mm.

At floral transition, the SAM starts bulging and then initiates a FM at the same time as a lateral IM is formed adjacently (Figure 2A). While the FM matures into a flower, the IM reiterates the process of forming one FM and initiating one lateral IM. This process generates a zigzag pattern because of a right angle shift at each successive FM initiation, occurring until activity ceases after production of approximately 6 to 8 flowers per inflorescence in our growing conditions (Figure 2BC).

In several mutants described here, meristems were observed that did not have a clear fate. Some meristems, which we dubbed “IM-like” (IML), looked like IMs but produced secondary meristems that did not mature to FM. In other cases, meristems formed in the inflorescence initiated leaves; these were recognised at an early stage by the triangular shape of the primordia they initiated and hence will hereafter be referred to as vegetative meristems (VM). For clarity of the text, we present the double mutants in two sets: those combining two mutations in genes that are expressed in the meristem, *S*, *FA* and *J* [26], [27], [29], and those obtained by crosses with *sft*, which is deficient in systemic florigenic protein [20], [21].

### The making of a *compound* inflorescence requires *J*

The inflorescence of the *s* mutant is highly branched and initiates many flowers (Figure 2D), as previously described [23], [29]. The formation of such a structure was due to the fact that at floral transition of the SAM, the first FM was replaced by an indeterminate IML which, like the IM, continued to produce other IMLs and hence participated in the branching of the inflorescence. The IMLs eventually formed flowers (Figure 2E), generating a highly branched, *compound* inflorescence. This phenotype was interpreted due to a delay in FM maturation and consequent extension of an indeterminate state, during which meristems proliferate [29].

Interestingly, when the *j* mutation was added to the *s* mutation, the inflorescence was not highly branched but looked like the inflorescence of the *j* mutant (compare Figure 2H with Figure 2D and Figure 2F) indicating that *j* was epistatic to *s*. The *j* and *j∶s* double mutants indeed produced inflorescences that initiated few flowers, usually 2 or 3, before reverting to vegetative functioning (Figure 2FH). This result suggests that *J* function is necessary to delay maturation of FM and/or extend IML fate in *s* single mutant. In *j* and *j∶s* the first steps of inflorescence formation were normal, but after initiation of a few FMs, a VM was visible in the normal location of the IM (Figure 2G). Very often, the VM produced one leaf, followed by alternation of few flowers and leaves (as was probably the case in Figure 2I) and finally, complete reversion to VM occurred [23].

### No *FA*, no flower

The *fa* mutant is characterized by the production of branched leafy inflorescences that contain no flowers, but accumulate meristems forming clumps of IMLs (Figure 2JK) [27], [28]. At floral transition of the SAM, the first FM was replaced by an indeterminate IML, which like the IM, continued producing other IMLs; some of these finally reverted to VM and initiated leafy shoots within the inflorescence (Figure 2L). The *fa* mutation also prevented flower formation in the compound inflorescence of *s*. The *fa* mutant and *s∶fa* double mutant produced branched and leafy inflorescences which were not distinguishable from each other (compare Figure 2JKL with Figure 2MNO), indicating that *fa* was epistatic to *s*.

By contrast, when *j* mutation was introduced in *fa* background, the inflorescence still contained leafy shoots but lacked the clumps of IMLs (Figure 2PQ), suggesting that the latter had their fate modified by the *j* mutation and that both mutations had additive effects. At floral transition of the SAM, in the *j∶fa* double mutant, two IMLs were formed which then created other IMLs as in *fa* but these meristems did not accumulate and eventually reverted to VM (Figure 2Q). These observations indicate that *j* mutation promotes reversion of the *fa* inflorescence to vegetative functioning. As a consequence of IML replacement by VM in the *j∶fa* double mutant, the branching of the inflorescence was reduced in comparison to the *fa* single mutant (compare Figure 2P with Figure 2J).

### *J* and *FA* expression domains mirror meristematic territories fated to be IM or FM

**Figure 3:**
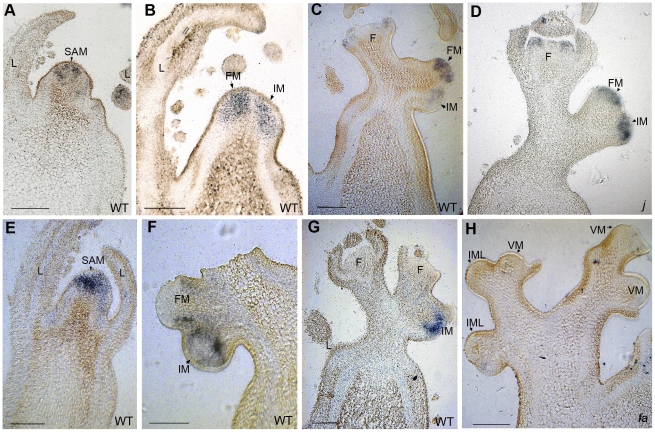
Detection of *FA* and *J* transcripts by *in situ* hybridization in longitudinal sections of tomato shoot apices. (A–C) *FA* expression at (A) vegetative, (B) transitional and (C) floral stages of Ailsa Craig WT. (D) *FA* expression in the inflorescence of the *j* mutant. (E–G) *J* expression in apices at (E) vegetative and (F–G) floral stages of WT. (H) *J* expression in the inflorescence of the *fa* mutant. F, flower; FM, flower meristem; IM, inflorescence meristem; IML, IM-like; L, leaf; SAM, shoot apical meristem; VM, vegetative meristem. Bars = 100 µm.

Both *j* and *fa* mutants formed leafy inflorescences, but the return to vegetative functioning occurred at different stages. In the *j* mutant, VM replaced IM after the initiation of few flowers (Figure 2G); in the *fa* mutant, VM were observed at positions which in the WT would give rise to individual flowers (Figure 2L). In order to examine whether these phenotypes are supported by expression patterns, we performed *in situ* hybridizations with *J* and *FA* probes on very young, WT inflorescences. Both genes were expressed in the SAM just before the transition to flowering (Figure 3AE), but at early stages of inflorescence formation, the expression of *FA* was progressively stronger in FM than in IM (Figure 3BC). This pattern was remarkably complementary to that of *J* which was stronger in IM than in FM (Figure 3FG). Differing from a previous study [26], we observed that the expression of *J* decreased during maturation of FM and was undetectable in young flowers (Figure 3G). By contrast, *FA* transcripts were present in sepal and petal primordia of young flowers (Figure 3C) as reported by Molinero-Rosales *et al.* [27].

The complementarity between the expression patterns of *J* and *FA* during inflorescence ontogeny suggested cross regulation between these genes. We therefore examined *FA* expression in *j* and observed that *FA* was expressed in all meristems of the mutant inflorescence (Figure 3D), suggesting that *FA* is repressed by *J* in the IMs of WT inflorescence. However it is worth noting that *j* inflorescence produced 2 or 3 flowers before reverting to vegetative functioning (Figure 2F) and hence the IM in the inflorescence sectioned in Figure 3D might be advanced towards FM maturation. We also examined *J* expression in *fa* mutant (Figure 3H). As described above, the inflorescences of *fa* were highly branched and accumulated IMLs (Figure 2J). We could not detect the expression of *J* in these IMLs, suggesting that these meristems had passed the stage of maturation when *J* was downregulated and thus that *FA* activity is not necessary for downregulation of *J*.

### Mutation in *SFT* increases inflorescence leafiness, whatever its architecture

**Figure 4:**
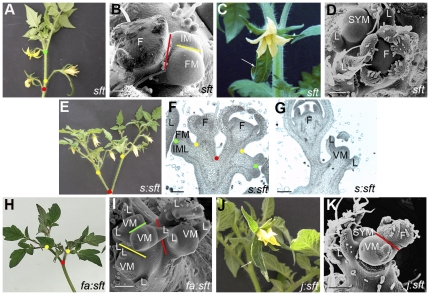
Inflorescence phenotype of tomato mutants. (A) and (B) Inflorescence of *sft* mutant showing several flowers; (C) and (D) Inflorescence of *sft* mutant with solitary flower; (E–G) *s∶sft* double mutant; (H) and (I) *fa∶sft* double mutant; (J) and (K) *j∶sft* double mutant. In microscopic pictures, colour bars show the clefts that occurred sequentially when new meristems were initiated to build-up the inflorescence. Red: 1^st^; yellow: 2^nd^, green: 3^rd^. Dots of same colours show the corresponding branching in macroscopic inflorescence picture. In C and J, arrows point at leaf-like sepals. Genotype abbreviations: *fa*, *falsiflora*; *j*, *jointless*; *s*, *compound inflorescence*; *sft*, *single flower truss.* Annotations: F, flower; FM, flower meristem; IM, inflorescence meristem; IML, IM-like; L, leaf; S, shoot; SYM, sympodial meristem; VM, vegetative meristem. Bars = 100 µm.

The *sft* mutant produced either inflorescences that reverted to vegetative growth after production of a variable number of flowers (Figure 4AB), or solitary flowers (Figure 4CD) as previously described [20]–[23]. When single flowers were produced, they showed leaf-like sepals (Figure 4C). This phenotype indicates that *SFT* is not necessary to make flowers, but might be involved in regulating IM fate.

When *sft* mutation was introduced into *s* background, we observed that the leafy phenotype of *sft* inflorescences was additive to the high branching character of *s*. The inflorescences of the *s∶sft* double mutant were definitively branched and contained flowers and leaves (Figure 4E). At the early stage of inflorescence initiation in the double mutant, two IMLs were formed that continued to initiate more IMLs, or eventually matured to FM as in the *s* mutant (Figure 4F). However, after production of some flowers, IMLs reverted to VMs and initiated leaves as in the *sft* mutant (Figure 4G). Lippman *et al.* observed a similar phenotype for the *s∶sft* double mutant [29].

Leafiness of the inflorescence was also increased in the *fa∶sft* double mutant as compared with the single *fa* mutant. *fa∶sft* produced branched leafy inflorescences that did not accumulate IMLs and never initiated flowers (Figure 4H). As in the *fa* mutant, two IMLs were produced at floral transition of the SAM; these IMLs produced a few more meristems that all reverted to VM (Figure 4I) so that the “leafiness” of the inflorescence was increased as compared to single mutant parents. Thus the inflorescence phenotypes of *sft* and *fa* were additive.

### Loss of *J* function increases the *single flower truss* phenotype of *sft*

Both the *j* and *sft* mutants showed reversion of the inflorescence towards vegetative functioning (Figure 2F and Figure 4A). In the *j* mutant, such a reversion did not modify the sympodial growth habit of the plants; reverted inflorescences still occupied a lateral position and successive inflorescences were regularly spaced on the shoot, as sympodial units. By contrast in the *sft* mutant, the architecture of the plant was more complex; several authors observed that the VM of the inflorescence might exert partial apical dominance over the presumptive SYM and so maintain a pole position as a “pseudoshoot” segment [21], [22]. In this pattern, each isolated flower appears as a *single flower truss* (explaining mutant's name) on the main, vertical axis of the plant which is in fact the vegetative inflorescence. Beside this phenotype, we observed that the *sft* inflorescence, albeit reverted to vegetative organogenesis, might occupy a lateral position, as in the *j* mutant (Figure S1). Both types of inflorescences could be observed on the same plants. We believe that this plasticity in the *sft* phenotype is environment-dependent since the frequency of the single flower phenotype was higher in limiting growing conditions (e.g. low light) [23].

The fact that *j* and *sft* mutations had similar effects on the architecture of the inflorescence suggested that *J* and *SFT* genes might have overlapping functions. Surprisingly, Shalit *et al.* reported that systemic SFT could restore the abscission zone in various mutants, although this was not tested with *j* [33]. To further analyze the relationship between *J* and *SFT*, we studied the double *j∶sft* mutant and observed that all plants produced solitary flowers which showed one or more enlarged, leaf-like sepal(s) (Figure 4J). This phenotype, which could also be observed in the single *sft* mutant as explained above (Figure 4C), was much more robust in the double *j∶sft* mutant. At floral transition, the SAM initiated a FM, but a VM occupied the position of the lateral IM (Figure 4K).

## Discussion

Initiation of a tomato inflorescence starts with simultaneous initiation of the first FM and a lateral IM (Figure 2A). A distinctive feature of IM compared to FM is that the former remains indeterminate and retains the ability to form other meristems, while the latter does not and matures into a flower. Tomato mutants showing defects in acquiring FM fate elaborate highly branched inflorescences and this led Lippman *et al.* to propose that progressive maturation of IM to FM defines a time window during which the next IM can be formed, to build-up the inflorescence [29]. This model explains that in *s* and *fa* mutants, the inflorescence is highly branched because FM maturation is delayed (in *s*) or blocked (in *fa*). During the ontogeny of such branched inflorescences, intermediate meristems accumulate which we dubbed ‘IMLs’. We find interesting to note that in the *s* and *fa* mutants, these IMLs and the inflorescence branches do not only form where the IM should be but also replace the FM, suggesting that in WT inflorescence, the first flower might derive from an IM that readily matures to FM.

We show in this paper that the MADS-box gene *J* is involved in the timing of FM maturation. The *j* mutation indeed suppressed the high branching phenotype of *s* and *fa* inflorescences (Figure 2), indicating that *j* mutation reduces the time window of FM maturation, otherwise extended by *s* or *fa* mutations. Consistently, acceleration of FM maturation in the inflorescence of the *j* mutant would explain that few flowers are formed, usually 2 or 3 as compared to 6 to 8 in WT. Interestingly, the acceleration of FM maturation does not generate a determinate inflorescence, but leads to its reversion to a vegetative program, the position of the lateral IM being then occupied by a VM in the inflorescence of *j* mutant (Figure 2G). Szymkowiak and Irish reported that this VM was completely suppressed when *blind* mutation - which compromises formation of axillary meristems in tomato [34] - was added to *j* and inferred from this observation that the reverted meristem of *j* leafy inflorescences is a sympodial meristem [26].

The function of the *J* gene in the WT inflorescence would thus be to prevent early maturation of FM. Such a function is obviously antagonistic with that of *FA*, which acts as a FM identity gene [27]. This antagonism is reflected in the complementary expression patterns observed between *J* and *FA* during early development of the inflorescence (Figure 3). We report here that although both genes were expressed in the SAM at the transition to flowering, their expression domains were distinct after the first FM and IM were formed: *J* was more strongly expressed in the IM than in the FM while the opposite was true for *FA*. These expression patterns suggested cross-regulation between *J* and *FA*. We therefore examined *FA* expression in *j* and observed that *FA* was expressed in all meristems of the mutant inflorescence. This “expansion” of *FA* supports our interpretation that *j* mutation accelerates FM maturation (see above), as well as our hypothesis that *J* represses *FA* in the IM of the WT inflorescence. By contrast, we could not detect *J* transcripts in the IMLs of the *fa* inflorescences, suggesting that *FA* activity is not necessary for downregulation of *J*. A good candidate for acting as a repressor of *J* would be *S* which is transiently expressed in the incipient IM before maturation to FM [29], but this hypothesis requires further investigation.

**Figure 5:**
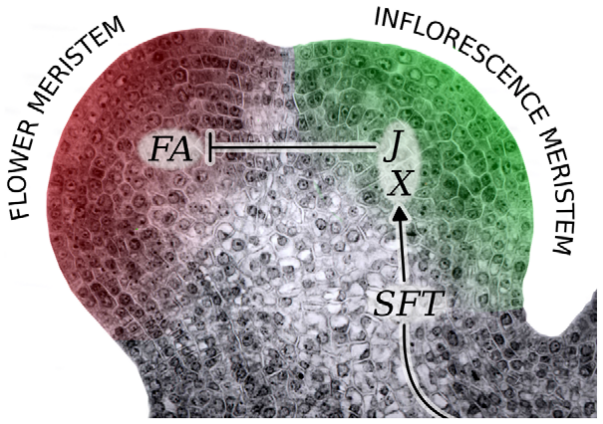
Hypothetical model of genetic interactions shaping the inflorescence of tomato. At floral transition, the SAM forms a flower meristem (FM) and a lateral meristem arises adjacently. *JOINTLESS* (*J*) and an unknown target (X) of the systemic SINGLE FLOWER TRUSS (SFT) protein prevent early FM maturation in the lateral meristem and so allow an inflorescence meristem (IM) fate. This involves repression of the FM identity gene *FALSIFLORA* (*FA*) by *J* in the IM.

At this stage we conclude from our study that *J* acts in the establishment of the IM by repressing the FM identity gene *FA*. But why, then, is the IM not completely lacking in the *j* mutant? It would certainly be expected that tomato plants impaired in acquiring IM fate would initiate single flowers whereas the *j* mutant elaborates inflorescences made of 2 or 3 flowers followed by leaves, and hence rather seem to be affected in maintenance of the IM as suggested before [23], [26]. By contrast, we report here that a very robust one-flowered inflorescence phenotype was obtained when *j* mutation was combined with *sft*, indicating that *J* and *SFT* cooperatively regulate the architecture of the inflorescence. Given that *J* is expressed in the meristem while *SFT* encodes a systemic signal, this suggests that J interacts with a meristematic target of SFT. Since *J* encodes a MADS-box protein of the SVP/AGL24 subfamily, good candidates for this target are other MADS-box proteins. Interactions were indeed found in the yeast two-hybrid system between J and MADS-box proteins from different subfamilies, represented by SOC1, AP1 and SEP [35] which in *Arabidopsis*, are targets of FT [3]. These findings lead us to propose that in tomato, J and a target of SFT (X) are involved in a protein complex repressing FM fate to allow establishment of an IM and branching of the inflorescence (Figure 5). Such a hypothesis is reminiscent of the inhibitory effect that SVP, AGL24 and SOC1 have together on flower differentiation in *Arabidopsis* [10], [11].

Although phylogenetic analyses indicate that *J* is the tomato ortholog of *SVP* [36], our results rather suggest that *J* fulfils in tomato the same function as the closely related gene *AGL24* in *Arabidopsis*: the promotion of IM fate. During inflorescence development, the expression pattern of *J* is indeed quite similar to that of *AGL24* in *Arabidopsis*, being more strongly expressed in IM than in FM [9] while expression of *SVP* is restricted to FM [37]. We also observed that the loss of *J* function suppressed the highly branched inflorescence phenotype of the *fa* mutant, just as the loss of *AGL24* rescues the inflorescence character of *lfy* mutants [9]. However, our study highlights a major divergence between the two species: in tomato, IM fate is established by *J* repressing FM fate (Figure 5) while inversely in *Arabidopsis*, *AGL24* must be repressed in the FM to suppress inflorescence identity [9]. This undoubtedly reflects the distinct ontogenic patterns of the inflorescences and suggests that *AGL24*-like genes might have a place in the genetic mechanisms underlying the diversity in inflorescence architecture [38].

## Materials and Methods

### Plant material and growth conditions

Seeds of the Ailsa Craig (AC; accession number LA2838A), Platense (Pl; accession number LA3243) and Rheinlands Rhum (RR; accession number LA0535) tomato cultivars and of the *s* (accession number LA3181; background AC), *sft* (accession number LA2460; background Pl) and *fa* (accession number LA0854; background RR) mutants were obtained from the Tomato Genetics Resource Center (University of California, Davis, U.S.A.). These alleles of *s*, *sft* and *fa* mutants have been previously described [22], [23], [27], [29]. The Heinz (Hz) cultivar and its isogenic *j* mutant were provided by the INRA (Institut National de la Recherche Agronomique, Montfavet, France). Hz is a determinate cultivar which is mutated for *SELF PRUNING* (*SP*) so that the *j* mutant in the Hz background is actually a double *j∶sp* mutant, that was originally described by [39]. The single *j* mutant was obtained by crossing *j*∶*sp* and AC plants. The *j* mutation was verified and was a large deletion as described by [24]. Seed stocks were made after several rounds of selfing in a glasshouse.

Seeds were germinated in a mix of peat compost brill (85%) and clay (15%) at 20°C. After two weeks, seedlings were transplanted into 7 cm×7 cm pots filled with a mix of peat compost brill (75%) ∶ clay (15%) ∶ perlite (10%). When 6-week old, plants were transplanted into larger pots (16 cm×16 cm) filled with the same substrate. Experiments were carried out either in a glasshouse in Louvain-la-Neuve (50°40′N 04°30′E) or in a growth cabinet in Liège (50°34′N 5°34′E). The glasshouse was heated and extra lighting was provided by PHILIPS HPLR 400 W bulbs to expose plants to a 16-h daylength and a minimum photon flux density of 100 µmol.m^−2^.s^−1^ (PAR) at the top of the canopy. For growth cabinet experiments, conditions were: 16-h daylength, 100–120 µmol m^−2^ s^−1^ (PAR) at leaf canopy level (V.H.O. Sylvania fluorescence tubes), 20°C, 70% relative humidity. Plants were watered daily with tap water and fed every two weeks with 12-12-17 N-P-K fertiliser (Compo, Benelux N.V.).

### Double mutant production and genotyping

The *s∶sft* and *s∶fa* double mutants were produced by crossing the *s* mutant as female parent and the *sft* mutant or a heterozygote *Fafa* plant as male parent, respectively. The *j∶s* double mutant was produced by crossing *j∶sp* mutant as female parent and *s* mutant as male parent. The *j∶fa* and *j*∶*sft* double mutants were produced by crossing the *j* mutant as female parent and either *FAfa* plant or s*ft* mutant as male parent, respectively. The *fa*∶*sft* double mutant was produced by crossing heterozygous *FAfa* plant as female and *sft* mutant as male parent. The F1 generation was self-fertilized and double mutants were identified in the F2 generation (following a 9∶3∶3∶1 mendelian segregation). Backcrosses were performed between the double mutant and their parental genotypes for *j∶s*, *s∶sft* and *j∶sft* to ascertain the presence of both mutations.

Since homozygous *fafa* plants were sterile, the *ss*∶*FAfa*, *jj*∶*FAfa*, *FAfa∶sftsft* mutants were selfed and their progeny was genotyped for *FA* alleles by PCR.

The *SP*, *S* and *FA* alleles were genotyped by PCR using a CAPS marker for *sp* [34], a dCAPS marker for *s* and spanning a 16-bp deletion in *fa* [27]. DNA was extracted according to [40]. For specific amplification, we used GoTaq DNA polymerase (Promega Benelux b.v), 50–100 ng of plant DNA as template. Primers were: 5′-ACCCCTTGTGATTGGTAGAGTG-3′ and 5′AGTGCCTGGAATGTCTGTGAC-3′ for *SP* (accession number U84140), 5′-CAAATTCGTATACTTGAAGCAATCTTTAATTCCAG-3′ and 5′-TGAATCCTGGAAGCAAAACC-3′ for *S* (accession number FJ190664), 5′-GATTATCGGAGGAACCAGTGCAG-3′ and 5′-ATTCCTCCACCTCCACCTCCTTGG-3′ for *FA* (accession number AF197934). The PCR conditions were: 2 minutes at 94°C, 35 cycles each consisting of 30 seconds at 94°C, 30 seconds at 60°C (for *SP* and *FA*) or 55°C (for *S*) and 1 minute at 72°C, then a final extension at 72°C for 5 minutes. The *SP* and *S* PCR products were digested with ScrFI and BstNI (new England Biolabs inc., Frankfurt, Germany) respectively. The *sp* mutant allele gives a PCR fragment of 1.1 kb whereas the WT allele gives two fragments of 650 and 400 bp; the *s* mutant alleles gives a PCR fragment of 456 bp and the WT of 422 bp; the *fa* mutant allele gives a PCR fragment of 204 bp whereas the WT amplicon is 220-bp long.

### Flowering time analysis

The flowering time of the initial segment was evaluated by the number of leaves produced below the first inflorescence. Counting was stopped when leaf number exceeded 40. Three independent experiments were carried out for each double mutant and the corresponding single mutants and wild-type cultivar.

Normality tests were performed and no additional transformation of the raw data was required. ANOVA I (SAS 9.1 system for windows) was performed to evaluate genotype effects. Differences between means were scored for significance according to the Scheffe F-test.

### Scanning electron microscopy

Samples were fixed in 2.5% glutaraldehyde in 0.1 M phosphate buffer (pH 7.2). They were vacuum infiltrated for 10 min and kept at 4°C overnight. The fixed tissues were then dehydrated through an ethanol series (25%–100%) at 4°C. Samples were critical-point dried in carbon dioxide, then coated with platinum at 0.07 mbar in a Balzers Union SCD 040 sputter coater and observed in a JEOL scanning electron microscope (JSM-840A) at 20 kV.

### Histological sections

Samples were fixed in FAA (ethanol 70%: acetic acid: formaldehyde 18∶1∶1), dehydrated in a graded ethanol series, embedded in paraffin and sectioned at 5 µm. Serial longitudinal sections were stained with haematoxylin-fast green and observed with a light microscope.

### *In situ* hybridization

Preparation of samples and *in situ* hybridizations were performed as described in [41]. ^35^S-labelled RNA probes were prepared with full-length coding sequence of *FA* cDNA (kindly provided by Prof R. Lozano, University of Almeria, Spain) and a 460-pb fragment of *J* cDNA (accession number AF275345) which excludes the MADS-box (PCR amplification was performed with primers 5′AAATTCTTGAGAGGCGTAT-3′ and 5′-CATGGATTTGTTACTGATTC-3′ at an annealing temperature of 50°C). Autoradiographs were observed in light-transmission microscopy.

## Supporting Information

Figure S1 **Lateral inflorescences of** ***sft*** **(A) and** ***j*** **(B) mutants.** I: inflorescence; L: leaf; S: shoot.
(TIF)
