# Supplementary figures and images for: Repression of Floral Meristem Fate Is Crucial in Shaping Tomato Inflorescence
Source: PLoS One. 2012 Feb 7;7(2):e31096. doi: 10.1371/journal.pone.0031096 (PMC3274538; doi:10.1371/journal.pone.0031096)

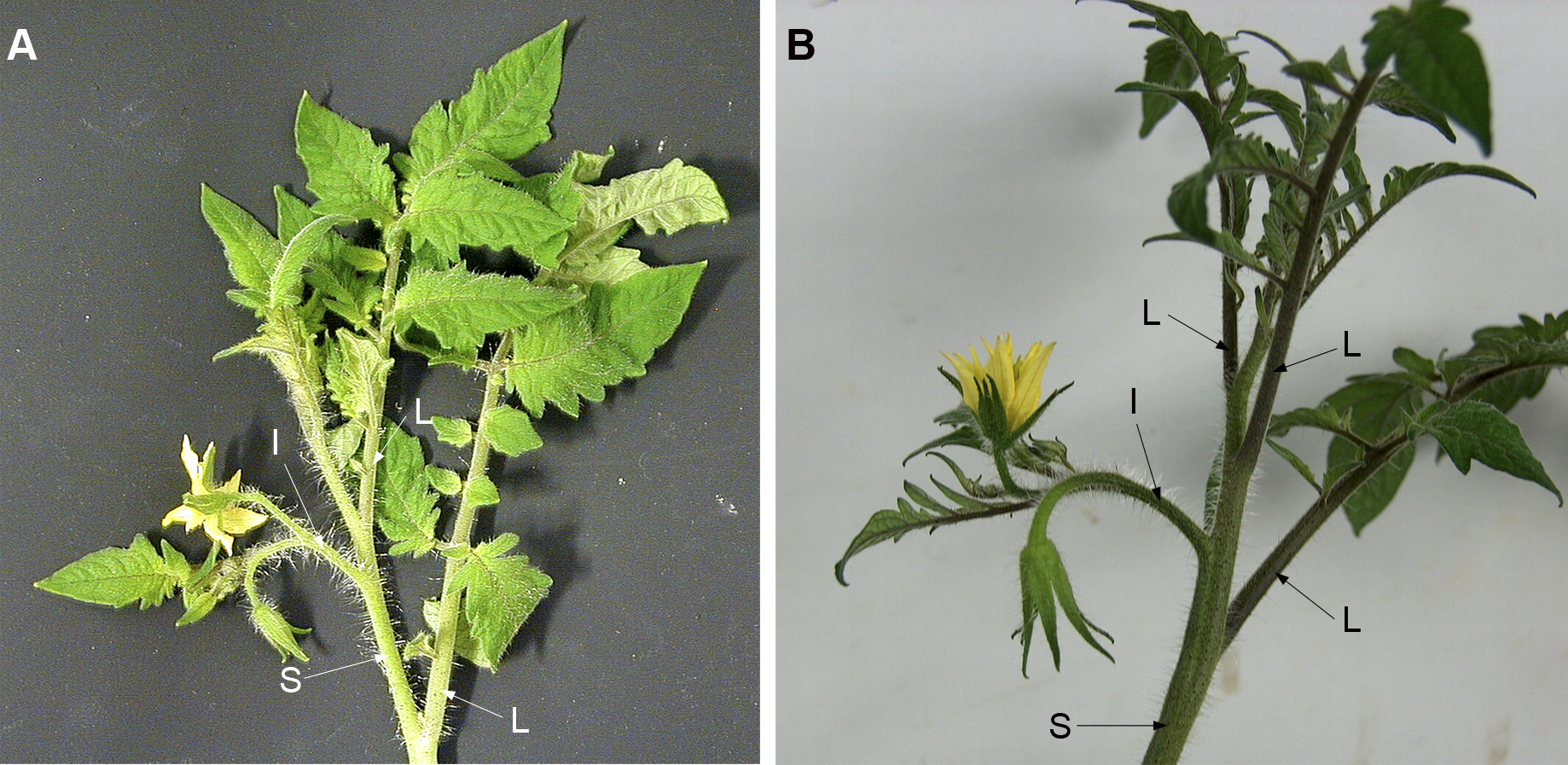

Supplement: Figure S1 — Lateral inflorescences of sft (A) and j (B) mutants. I: inflorescence; L: leaf; S: shoot. (TIF) [file pone.0031096.s001.tif]
